# Patient awareness of loss of protective sensation in the diabetic foot: an opportunity for risk reduction?

**DOI:** 10.1186/1757-1146-4-S1-P37

**Published:** 2011-05-20

**Authors:** Sylvia McAra

**Affiliations:** 1 School of Community Health, Podiatry Department, Charles Sturt University, Albury, NSW, 2640, Australia

## Background

Peripheral sensory neuropathy is a common complication in diabetes. Neuropathy is the single most powerful risk factor for diabetic foot amputation. Often foot care efforts are thwarted by the phenomenon of inadvertent injury, from events which may appear to the clinician as chronic and ignorant self-neglect on the part of neuropathic clients.  This study aimed to determine the proportion of a study population of people with diabetes and peripheral neuropathy who were simultaneously unaware of this sensory loss.

## Method

Thirty-two (32) people with symptoms of diabetic neuropathy were tested. Subjects aligned themselves with one of two groups. Group1 consisted of those who predicted that their protective sensation was intact, by indicating that they expected to be able to feel if a blister was forming on their foot, and Group 2 comprised those who expected not to be able to feel if a blister was occurring.  The presence or absence of protective sensation was determined by testing with the 5.07 (10 gram) Semmes Weinstein monofilament.

## Results

Seventy-eight percent (78%) of the study population (25 of 32 subjects) assumed that their protective sensation was intact, however only twenty-five percent (25%) of these (8 subjects) were correct in this assumption according to the test criteria. Fifty-three percent (53%) of the total study population (17 of 32 subjects) demonstrated a lack of protective sensation and a concomitant lack of awareness of this loss.

## Conclusion

This lack of awareness, even amongst clients previously assessed and educated in regard to their loss of protective pedal sensation, suggests an opportunity for more effective diabetes education programs to counter an important and potentially modifiable risk factor in diabetic foot ulceration and amputation. It is a fundamental concept for this “at risk” group to grasp in order for them to be able to best understand and manage their potential for inadvertent and unnoticed injury, and thereby to empower them to be more effective in their efforts to self care for their neuropathic feet.

